# Singlet fission initiating triplet generations of BODIPY derivatives through $\pi$-stacking: a theoretical study

**DOI:** 10.1038/s41598-022-23370-y

**Published:** 2022-11-16

**Authors:** Takao Tsuneda, Tetsuya Taketsugu

**Affiliations:** 1 Department of Chemistry, Faculty of Science, Hokkaido University, Sapporo, 060-0810 Japan; 2 Graduate School of Science Technology and Innovation, Kobe University, Nada-ku, Kobe, Hyogo 657-8501 Japan; 3 Institute for Chemical Reaction Design and Discovery (WPI-ICReDD), Hokkaido University, Sapporo, 001-0021 Japan

**Keywords:** Chemical biology, Chemistry, Energy science and technology, Physics

## Abstract

The role of singlet fission (SF) in the triplet-state generation mechanism of 1,3,5,7-tetramethyl-boron-dipyrromethene derivatives is revealed by exploring the cause for the solvent dependence of the generation rate. Comparing the adsorption energy calculations of solvent molecules, i.e., cyclohexane, chloroform and acetonitrile molecules, to the derivatives with the $\pi$-stacking energies of these derivatives surprisingly show that the hierarchy of the solvation energies and $\pi$-stacking energies strongly correlates with the experimentally-suggested solvent dependence of the triplet-state generation of these derivatives for five and more adsorbing solvent molecules. Following this finding, the excitation spectra of these derivatives in acetonitrile solvent are explored using the proprietary spin-flip long-range corrected time-dependent density functional theory. It is, consequently, confirmed that the $\pi$-stacking activates the second lowest singlet excitation to trigger the spin-allowed transition to the singlet doubly-excited tetraradical (TT)$_1$ state, which generates the long-lived quintet (TT)$_1$ state causing the SF. However, it is also found that the $\pi$-stacking also get a slow intersystem crossing active around the intersections of the lowest singlet excitations with the lowest triplet T$_1$ excitations in parallel with the SF due to the charge transfer characters of the lowest singlet excitations. These results suggest that SF initiates the triplet-state generations through near-degenerate low-lying singlet and (TT) excitations with a considerable singlet-triplet energy gap after the $\pi$-stacking of chromophores stronger than but not far from the solvation. Since these derivatives are organic photosensitizers, this study proposes that SF should be taken into consideration in developing novel heavy atom-free organic photosensitizers, which will contribute to a variety of research fields such as medical care, photobiology, energy science, and synthetic chemistry.

## Introduction

Singlet fission (SF) is an electronic state transition process that induces the singlet (S) to triplet (T) state transition through the electronic excitation and the singlet-quintet transition such as^1–3^

1 $$\begin{aligned} \hbox { S}\ _0 + \hbox { S}\ _1 \rightarrow ^1\text{(TT) }\rightarrow ^5\text{(TT) } \rightarrow 2\hbox { T}\ _1, \end{aligned}$$

where (TT) indicates the state consisting of two separated triplet states. Different from the intersystem crossing (ISC), SF rapidly proceeds even for heavy atom-free molecules, because it is a spin-allowed transition. Singlet fission attracts attentions as a probable mechanism for exceeding the Shockley-Queisser theoretical limit of efficiency of organic solar cells, 33.7%, toward 44.4%^4^. In principle, SF takes place in multiple chromophores when the triplet (T) excitation energy is less than the half of the lowest singlet (S$_1$) excitation energy, i.e.,

2 $$\begin{aligned} 2\Delta E\text{(T) }\lesssim \Delta E\text{(S }_1\text{) }, \end{aligned}$$

where $\Delta E$ indicates the excitation energy^1,5^. Singlet fission proceeds even if the double enthalpy of the generated triplet-state chromophores slightly exceeds the enthalpy of the initial singlet-state ones^6^ by seemingly violating the energy conservation principle^7^ due to the entropy enhancement by the diffusion of the generated triplet-state chromophores in the subsequent (TT)$\rightarrow$2T process^8^. Recently, it is reported that the doubly-excited tetraradical S$_1$ state, i.e., singlet $^1$(TT) state, is rapidly converted to the quintet $^5$(TT) state^2,3^. The energy difference of these states is theoretically evaluated to be negligible for $\pi$-stacking chromophores: only about 10 meV in tetracene dimer^9^. This indicates that SF can be energetically discussed using the $^5$(TT) excitations. Then, according to a time-resolved electron spin resonance spectroscopy study, the $^1$(TT) and $^5$(TT) states are mixed with each other even in the absence of spin-orbit coupling^10^ during the first 100–500 ns in bridged bipentacenes, and then decay to the ground state by concerted recombination or to two T$_1$ states by splitting^2^. The transition of $^1$(TT) to $^5$(TT) states is interpreted to take place by the zero-field splitting interaction at the singlet-quintet level-crossing in the presence of negative exchange coupling during the triplet-exciton diffusion and subsequent re-encounter in the highly disordered region^11^. Note that since this re-encounter needs, at least, double (TT) dimers with four chromophores, the lifetime of the triplet-state chromophores significantly depend on the concentration of chromophores^6^. The lifetime of the singlet state is investigated for bis(triisopropylsilylethynyl) (TIPS) pentacene, a solar cell material, after the SF^6^. Consequently, it is found that the yield and lifetime of the produced triplet state increase as the concentration enlarges. Based on the transition mechanism of the $^5$(TT) state^11^, this indicates that the diffusions of triplet states prolong the lifetime of SF through generating the $^5$(TT) states without photoirradiation. Note that singlet-fission systems are mostly polymers containing benzene rings like oligoacenes or ethylene units like polyenes, all of which form strong $\pi$ stackings^1^. This implies that SF usually proceeds after the $\pi$-stacking of chromophores, and therefore, it is dependent on the concentration of chromophores.

However, recent theoretical and experimental studies have raised questions about the contribution of SF to the initiation of triplet generations in boron-dipyrromethene (BODIPY) derivatives^12,13^. Based on *ab initio* complete active space self-consistent field (CASSCF) calculations, Duman et al. obtained that 8,8’- and 8,2’-bis-tetramethyl (TM) BODIPY (bis-TMBODIPY) dyes, in which two TMBODIPY molecules are bonded at (8,8’) and (8,2’) positions, possess the S$_1$ state of doubly-excited configurations, which are not given in the configurations of the monomer^14^. As a result, they suggested that the $^1$(TT)$_1$ state is supposed to contribute to the spin-orbit coupling with the T$_1$ state, implying that SF leads to the ISCs. Montero et al. traced the generation process of the triplet states of 8,2’-bis-TMBODIPY (BODIPY 546) by femtosecond and nanosecond transient absorption measurements^15^. Consequently, they found that the triplet states are generated in a few picoseconds with transitory efficiencies more than 100% and that the generated triplet states, which correspond to the absorption band around 413 nm, have very long lifetime even in the presence of oxygen at low concentration. Based on these findings, they concluded that BODIPY 546 also uses SF in the triplet generation mechanism. Michl and coworkers, however, theoretically countered this conclusion^13^. By performing highly-accurate complete active space second-order perturbation (CASPT2) calculations, they showed that the above-mentioned $^1$(TT)$_1$ state of bis-TMBODIPY is an artifact state coming from the imbalanced active space of the CASSCF calculation and suggested that SF do not occur for this bis-dimer due to the violation of Eq. (2) in the CASPT2(16,16) calculations. Kandrashkin et al. then performed the time-resolved electron paramagnetic resonance spectroscopy analysis of a bis-TMBODIPY^16^. As a result, they found that electron polarization patterns are different for two TMBODIPY subunits and that no above-mentioned quintet states is detected in this spectroscopy. They, therefore, concluded that SF is excluded from the probable triplet-state generator for this bis-TMBODIPY. Following these counterarguments, Montero et al. recently performed the femtoseconds transient absorption measurements of this BODIPY 546 and other two TMBODIPY derivatives with acceptor and donor groups (8-*p*-nitrophenyl- and 8-*p*-aminophenyl-TMBODIPYs)^12^. As a result, they found that these TMBODIPY derivatives possess triplet generations sensitively depending on the solvents: the triplet state generation rapidly proceeds in acetonitrile but it progresses very slowly in cyclohexane, while it is fast for the 8-*p*-nitrophenyl-TMBODIPY but it is very slow for the 8-*p*-aminophenyl-TMBODIPY in chloroform. They concluded that the triplet state generation of these chromophores comes from not the SF but ISC through the formation of a charge transfer (CT) state, on the basis of this sensitive solvent dependence. However, the CT state, which appears only in polar solvent and contributes to the ISC, is not specified in this study. The timescales of the main triplet generations, single-digit pico seconds, are also too short for spin-forbidden spin-orbit transitions of such heavy atom-free systems to proceed, while those of the secondary triplet generations, single-digit nano seconds, are appropriate as the timescale of the ISC. Note that conventional studies including this study have, so far, disconfirmed only the SF contributions to the intramolecular SF of bis-TMBODIPYs. It is, therefore, still an open question whether SF contributes to the triplet-state generation for other BODIPY derivatives like these TMBODIPY derivatives.

In this study, we explore the contribution of SF to the triplet-state generation of two $\pi$-stacking TMBODIPY derivative dimers by investigating the solvent effect on the dimerization and the excitations of the dimers for various spin multiplicities, following the study of Montero et al.^12^. Based on this study, we make clear the cause for the solvent-dependence of the triplet-state generation and theoretically explore the SF contribution to their high triplet-state generation rates despite of their heavy atom-free structures.

## Methods

**Figure 1:**
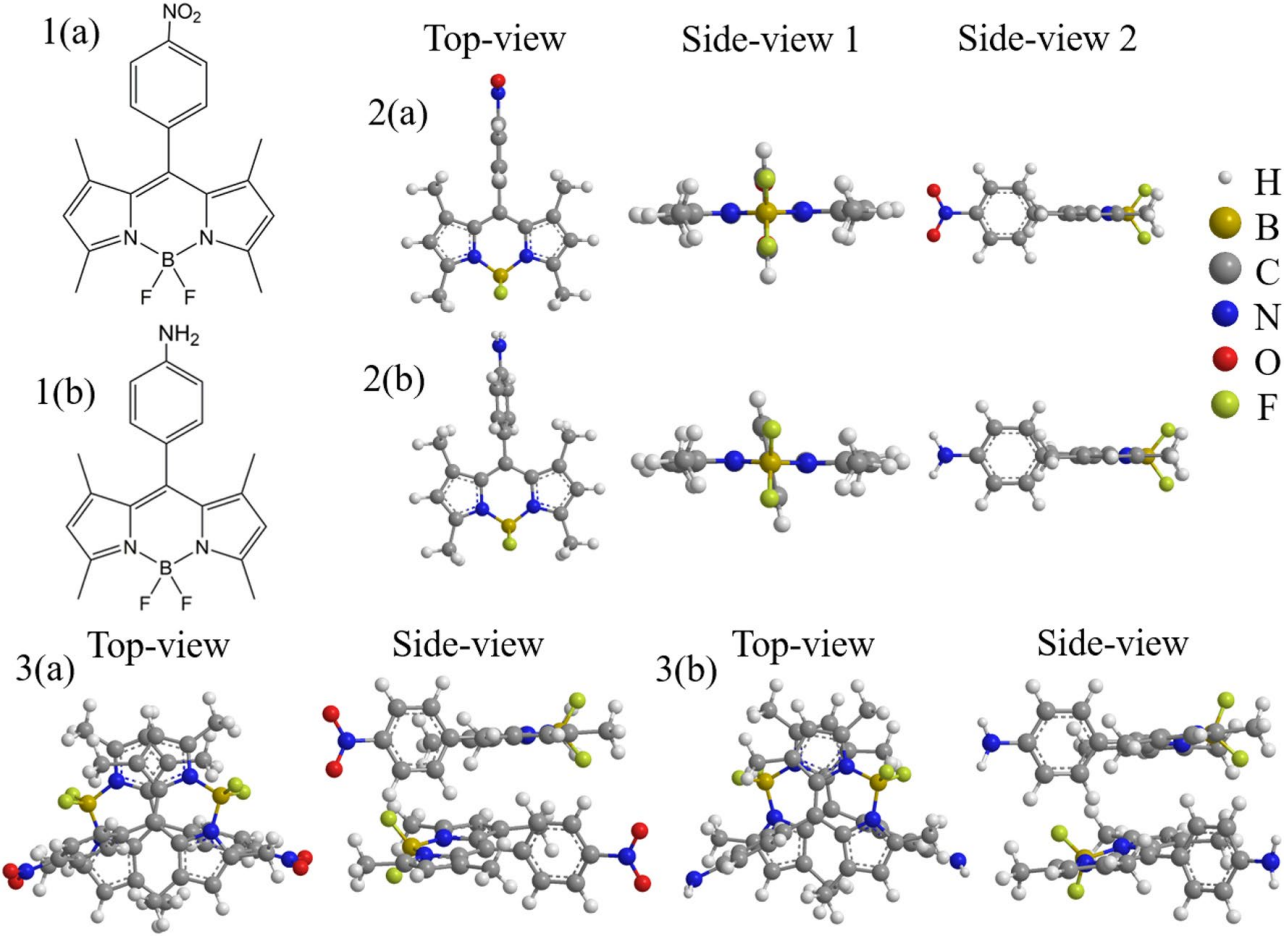
Optimized structures of TMBODIPY derivatives (**a**) and (**b**) monomers and their $\pi$-stacking dimers including the solvent effect of acetonitrile.

**Figure 2:**
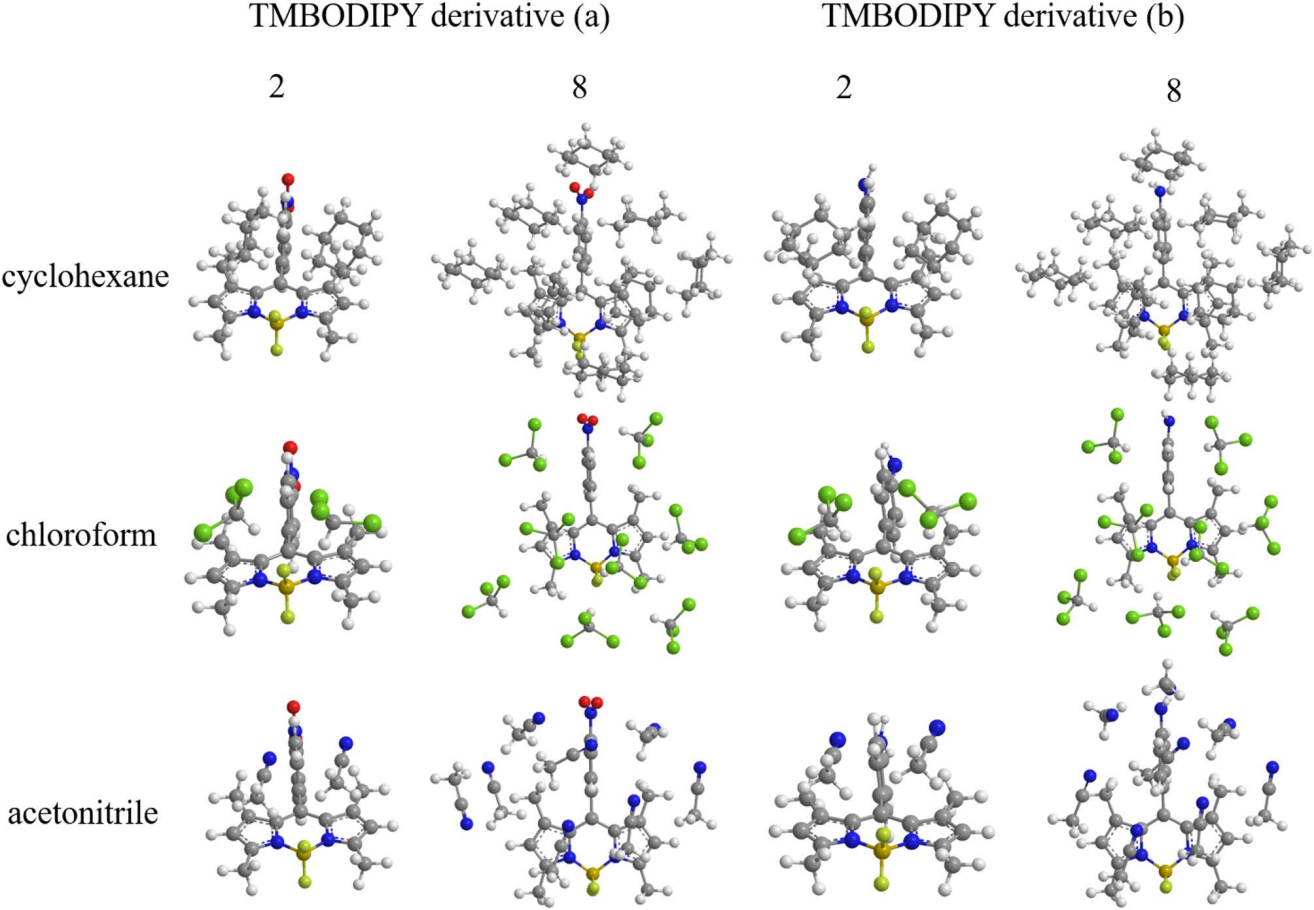
Optimized structures of TMBODIPY derivatives (**a**) and (**b**) monomers adsorbing two and eight solvent molecules to the single side in the monolayers for three types of solvent molecules: cyclohexane, chloroform and acetonitrile. For each adsorption, the corresponding solvent effects are incorporated by CPCM.

Long-range corrected (LC) Kohn-Sham density functional theory (DFT)^17,18^, LC-time-dependent (TD) DFT^19,20^ and collinear spin-flip LC-TDDFT^21,22^ calculations are performed using the cc-pVTZ basis set^23^ for TMBODIPY derivatives, (a) 8-*p*-nitrophenyl TMBODIPY and (b) 8-*p*-aminophenyl TMBODIPY, and their $\pi$-stacking dimers, which are shown in Fig. 1. The structures of TMBODIPY monomer adsorbing one through eight solvent molecules are also calculated for the solvents of cyclohexane, chloroform and acetonitrile, as illustrated only for two and eight adsorbing molecules in Fig. 2 (For other adsorption structures, see Fig. S1 in the supporting information). The long-range correction^24,25^ for the Becke 1988 exchange^26^ + Lee-Yang-Parr correlation^27^ (LC-BLYP) functional is used in these calculations, though $\omega$B97XD^28^ dispersion-corrected LC functional is employed only in the geometry optimizations of the dimers. Note that the spin-flip LC-TDDFT is one of the most sophisticated TDDFT including both long-range exchange and doubly-excited configuration correlation, and has been established to provide very accurate excitation energies for many systems^29,30^, while $\omega$B97XD contains both long-range exchange and dispersion correlation effects, which are required to quantitatively calculate $\pi$-stacking energies. Actually, it is reported that conventional TDDFT underestimates the triplet excitation energies, while it overestimates the singlet excitation energies^31^. As far as we know, the spin-flip LC-TDDFT is the only TDDFT that can solve this problem. The solvent effect is included by the conductor-like polarizable continuum model^32^ of cyclohexane, chloroform and acetonitrile. Geometry optimizations are carried out for the S$_0$ and T$_1$ excitations for the monomers and the S$_0$ and $^5$(TT)$_1$ excitations for the dimers. In the geometry optimizations, we examined several initial adsorption structures to search for the most stable structures. The solvent molecules are arranged to adsorb the molecular plane of BODIPY with keeping away from the nitrophynyl and aminophenyl groups as much as possible. For the optimized adsorption structures, see Fig. S1 of the supporting information. The TDDFT calculations are carried out using the optimized geometries of the S$_0$ and T$_1$ excitations for the monomers and dimers. DFT and TDDFT calculations are performed with the Gaussian 16 Revision A.03 program^33^, while spin-flip TDDFT calculations are carried out with the development version of GAMESS program^34^.

## Results and discussions

### Adsorption energies of solvent molecules versus $\pi$-stacking energies

In the femtosecond transient absorption measurements of TMBODIPY derivatives (a) and (b), Montera et al.^15^ interpret that the dependence of the triplet-state generation on the solvent polarity is attributed to the appearance of a CT excited state, which causes the ISC to a triplet state following the El-Sayed rule^35^. However, it has been revealed neither the CT state leading to the ISC nor the cause for the dependence of the triplet-state generation on the difference between the derivatives (a) and (b). The $\pi$-stacking of chromophores are presumed to initiate SF as mentioned in Sect. “Introduction”. On the other hand, strong $\pi$-stacking hinder the dissociation of the triplet-state chromophores after the quintet (TT) generations. It is actually known that indigo satisfying Eq. (2) induces not SF but ISC due to the large $\pi$-stacking energy leading to the crystalization^36^. Consequently, $\pi$-stacking energy should be higher than but not far from the solvation energy to induce SF. Comparing the $\pi$-stacking energy with the solvation energy, therefore, elucidates the feasibility of SF.

**Figure 3:**
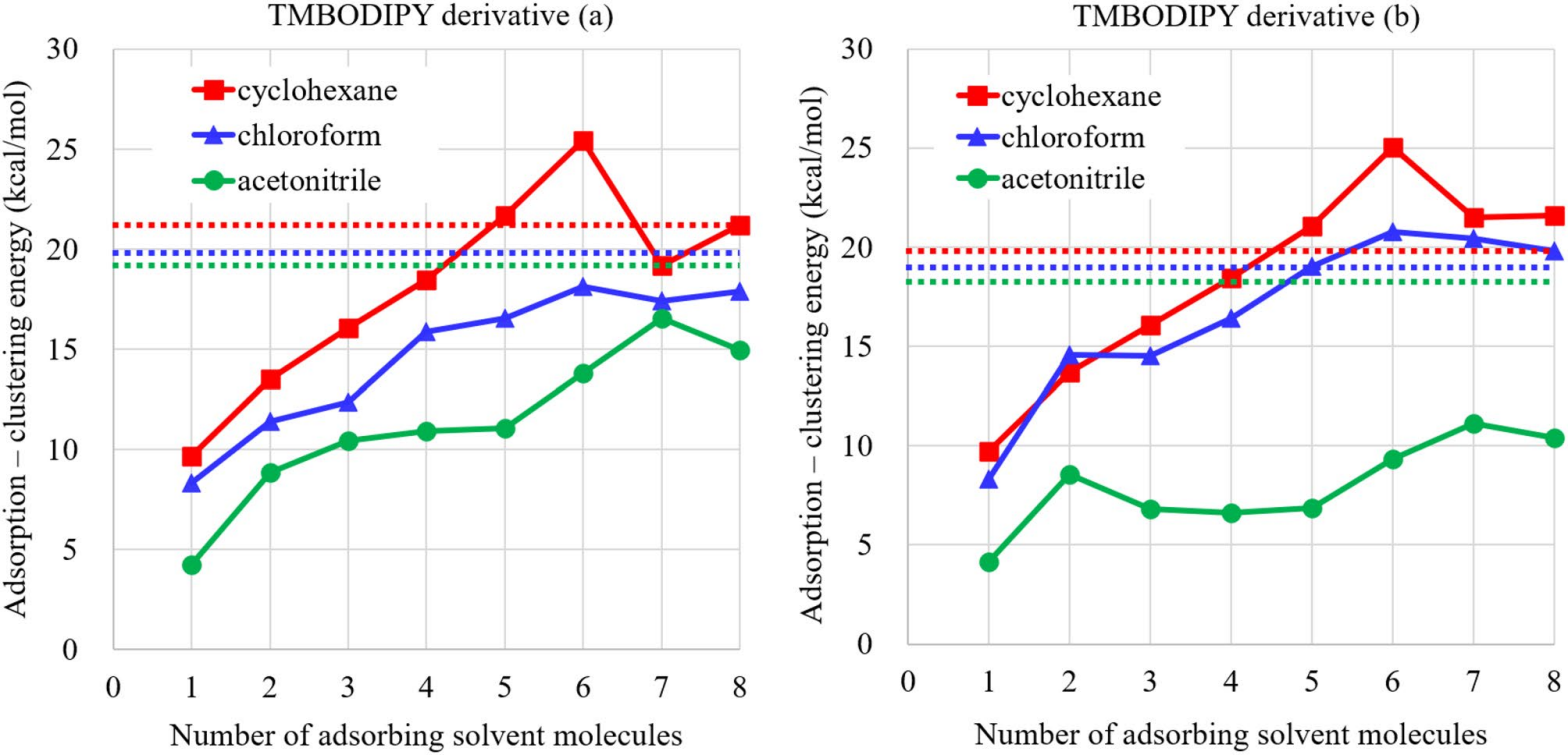
Adsorption energies of monolayer cyclohexane, chloroform and acetonitrile solvent molecules on the single sides of TMBODIPY derivatives (**a**) and (**b**) monomers, from which the clustering energies of the solvent molecules are subtracted, in terms of the number of the adsorbing solvent molecules. Calculations are performed by $\omega$B97XD/cc-pVTZ method with the CPCM solvent effect corresponding to the adsorbing solvent molecules. The $\pi$-stacking energies of TMBODIPY derivatives (**a**) and (**b**) dimers in cyclohexane, chloroform and acetonitrile are illustrated as red, blue and green dotted lines, respectively.

We, therefore, first explore the solvent effects of the $\pi$-stacking dimers. In solvent, $\pi$-stackings proceed only when the sum of the adsorption energies of the neighboring solvent molecules is lower than the $\pi$-stacking energy. Note that since the solvent molecules form their cluster structures, the clustering energies of the solvent molecules should be subtracted from the $\pi$-stacking energy. Figure 3 plots the adsorption energies of cyclohexane, chloroform and acetonitrile solvent molecules to the single sides of TMBODIPY derivatives in monolayers, from which the clustering energies (Fig. S2 of the supporting information) are subtracted, in terms of the number of the adsorbing solvent molecules.

Note that the dotted lines indicate the $\pi$-stacking energies of TMBODIPY derivative (a) and (b) dimers for each solvent. Surprisingly, it is found that the adsorption energies of chloroform molecules are lower than the $\pi$-stacking energy for the derivative (a), while they exceed the $\pi$-stacking energy for the derivative (b) adsorbing six and more chloroform molecules. This indicates that in chloroform solvent, the derivative (a) forms the $\pi$-stacking structure, while the derivative (b) keeps on solvating. On the other hand, both derivatives provide larger adsorption energies of cyclohexane molecules than the $\pi$-stacking energies, while they give smaller adsorption energies of acetonitrile molecules than the $\pi$-stacking energies. This indicates that both derivatives hardly form the $\pi$-stackings in cyclohexane solvent, while they rapidly form the $\pi$-stackings in acetonitrile solvent. These results are clearly consistent with the experimental findings^15^. The experimental results show that the triplet-state generation rapidly proceeds for both derivatives in acetonitrile solvent and it rapidly occurs only for the derivative (a) in chloroform solvent, while it takes place very slowly for other combinations of derivative and solvent. Therefore, the triplet-state generation of the TMBODIPY derivatives correlate with the feasibility of the $\pi$-stacking in each solvent. This result clearly indicates that the triplet states are generated through the $\pi$-stacking and that SF contributes to the triplet-state generation of the TMBODIPY derivatives in contrast to conventional discussions for this experimental result.

### Excitations of the monomer and $\pi$-stackings dimer of TMBODIPY derivative (a)

**Figure 4:**
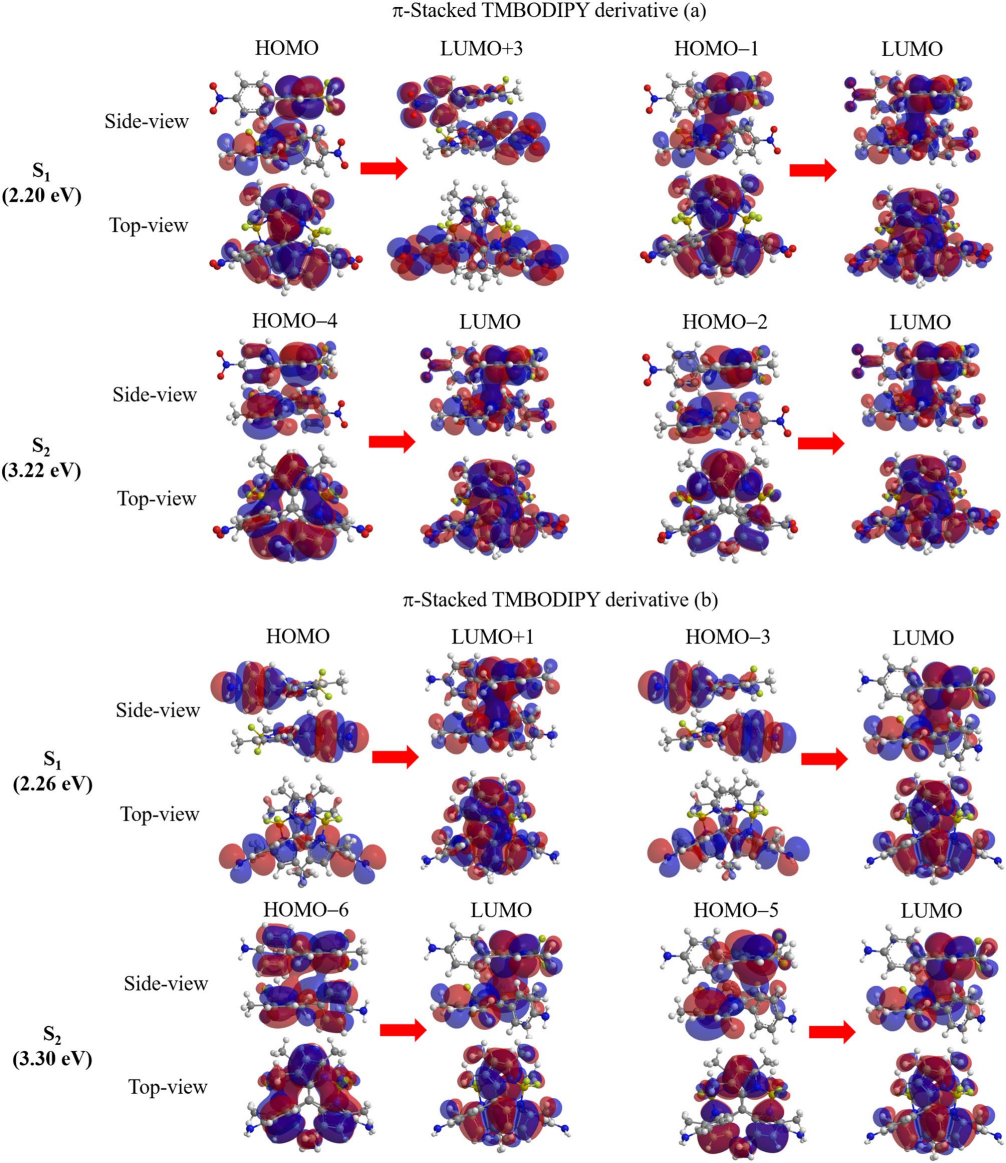
Molecular orbital images corresponding to the main transitions of the S$_1$ and S$_2$ excitations of $\pi$-stacking TMBODIPY derivatives (**a**) and (**b**) dimers in acetonitrile solvent. The transitions are given in SF-LC-TDBLYP/cc-pVTZ calculations, while the molecular orbital images are obtained in LC-BLYP/cc-pVTZ calculations .

To explore the contribution of SF, we calculated the excitation energies of TMBODIPY derivatives (a) and (b) for the monomers and $\pi$-stacking dimers in acetonitrile solvent. Table 1 compares the calculated excitation energies of TMBODIPY derivative (a) to the experimental values. For fairness, the experimental spectral peak energy of the S$_1$ excitation is listed in the columns of both the monomer and dimer^12^. The table shows that for both the monomer and dimer, spin-flip LC-TDDFT (SF-LC-TDBLYP) underestimates the S$_1$ excitation energies by 0.26 or 0.27 eV, while LC-TDDFT (LC-TDBLYP for the monomer and TD$\omega$B97XD for the dimer) overestimates them by 0.50 and 0.29 eV, respectively. Figure 4 displays the molecular orbitals corresponding to the main transitions of the S$_1$ and S$_2$ excitations in acetonitrile solvent, which are obtained by spin-flip LC-TDDFT calculations (for the MOs of S$_3$ and S$_4$ excitations, see Fig. S4 in the supporting information). As clearly shown in the figure, the main transition of the S$_1$ excitation, HOMO to LUMO+3 transition, is a CT transition from the TMBODIPY to the nitrophenyl group. Note also that the second transition of the S$_1$ excitation of the monomer, HOMO to LUMO+1 transition, is also a similar CT transition (see Fig. S3 of the supporting information). For cyclohexane, these CT transitions are obtained in the same excitations. These CT transitions may enhance the feasibility of ISC to a triplet state according to the El-Sayed rule^35^, as the experiment suggests^12^. However, we should notice that the T$_1$ excitation energy of the $\pi$-stacking dimer (1.55 eV) is much lower than the S$_1$ one (2.20 eV), while the T$_2$ excitation energy (2.98 eV) is calculated to be even higher than the S$_1$ one (2.76 eV). Similarly, the T$_1$ excitation energy of the monomer (1.44 eV) is much lower than the S$_1$ excitation energy (2.97 eV). These results suggest that ISC proceeds through the S$_1$ $\rightarrow$T$_1$ transition in both the monomer and dimer of the derivative (a), though it is very slow compared to the SF. Assuming that SF proceeds, the experimental peak value of the CT band is naturally attributed to the $^1$(TT)$_1$ excitation of the $\pi$-stacking dimer: As shown in the table, the calculated $^5$(TT)$_1$ excitation energy (2.76 eV), which is supposed to approximate the $^1$(TT)$_1$ excitation energy, is close to the CT band energy (2.99 eV). The table also shows that the dimer gives large oscillator strengths *f* for both the S$_1$ and S$_2$ excitations in contrast to the monomer providing large *f* values only for the S$_1$ excitation. Note that the SF does not necessarily take place from the S$_1$ state, because it initially proceeds through the spin-allowed transition to the $^1$(TT)$_1$ state. The $^5$(TT)$_1$ excitation of the dimer lies between the S$_1$ and S$_2$ excitations with the energy of 0.48 eV below that of the S$_2$ excitation. As mentioned in Sect. “Introduction”, it is reported that the $^1$(TT)$_1$ excitation energy is very close to the $^5$(TT)$_1$ one^9^. Note that highly-lying excited state-mediated SF processes are recently reported for perylene^37^ and C$_\mathrm{2h}$ skeletons^38–40^. This result, therefore, indicates that SF proceeds from the S$_2$ excitation for the derivative (a).

**Table 1:** Calculated vertical excitation energies (eV) of TMBODIPY derivative (a) for the monomer and $\pi$-stacking dimer by spin-flip LC-TDBLYP, LC-TDBLYP (monomer) and TD$\omega$B97XD (dimer) with cc-pVTZ basis sets and CPCM solvent effect of acetonitrile.

Excited state	Exp.^a^		SF-LC-TDBLYP			LC-TDBLYP/TD$\omega$B97XD		
	eV	nm	Main transitions	eV	nm	eV	nm	*f*
**Monomer in acetonitrile**								
T$_1$				1.54	806	1.44	861	
S$_1$	2.47	501	$$\left\{ \begin{array}{l} \text{ H }\rightarrow \text{ L }~(-0.71) \\ \text{ H }\rightarrow \text{ L+1 }~(0.69) \end{array} \right. $$	2.21	562	2.97	418	0.5804
S$_2$			$$\left\{ \begin{array}{l} \text{ H }-1\rightarrow \text{ L }~(-0.86) \\ \text{ H }\rightarrow \text{ L }~(-0.29) \end{array} \right. $$	3.30	376	4.01	309	0.0880
$\user2{\pi }$**-Stacking dimer in acetonitrile**								
T$_1$				1.55	802	1.38	899	
$^5$(TT)$_1$	2.99	415				2.76	449	
S$_1$	2.47	501	$$\left\{ \begin{array}{l} \text{ H }\rightarrow \text{ L+3 }~(0.68) \\ \text{ H }-1\rightarrow \text{ L }~(0.65) \end{array} \right. $$	2.20	564	2.76	450	0.1194
S$_2$			$$\left\{ \begin{array}{l} \text{ H }-4\rightarrow \text{ L }~(0.49) \\ \text{ H }-2\rightarrow \text{ L }~(0.45) \end{array} \right. $$	3.22	385	2.93	423	0.9479
S$_3$			$$\left\{ \begin{array}{l} \text{ H,H }\rightarrow \text{ L,L }~(0.98) \\ \text{ H,H }\rightarrow \text{ L,L+1 }~(-0.12) \end{array} \right. $$	3.34	371	3.69	336	0.0158
S$_4$			$$\left\{ \begin{array}{l} \text{ H }-5\rightarrow \text{ L }~(-0.69) \\ \text{ H }-4\rightarrow \text{ L }~(-0.49) \end{array} \right. $$	3.54	351	3.69	336	0.0052

^a^Ref.^12^.

Main transitions of spin-flip LC-TDBLYP excitations are also shown with the coefficients of the response functions in parentheses, in which the notations H and L indicate HOMO and LUMO. The oscillator strengths (*f*) of LC-TDBLYP (monomer) and TD$\omega$B97XD (dimer) are also listed.

These results reveal the SF mechanism of the derivative (a): The S$_2$ excitation with a high oscillator strength is stabilized through the structural relaxation toward the intersection with the $^1$(TT)$_1$ excitation. After the spin-allowed transition to the $^1$(TT)$_1$ state, zero-field splitting interaction in the presence of negative exchange coupling during triplet-exciton diffusion and subsequent re-encounter in the highly disordered region spontaneously generates a long-lived mixing state with the $^5$(TT)$_1$ state^11^. The dimer in the $^5$(TT)$_1$ state is then split into two monomers in the T$_1$ state. In parallel with the SF process, the ISC to the T$_1$ state proceeds very slowly from the S$_1$ state of the CT character in both the monomer and $\pi$-stacking dimer.

### Excitations of the monomer and $\pi$-stackings dimer of TMBODIPY derivative (b)

Let us next look into the contribution of SF to TMBODIPY derivative (b). Table 2 compiles the calculated excitation energies of TMBODIPY derivative (b). Similar to the results of the derivative (a), LC-TDDFT tends to slightly overestimate the excitation energies by 0.39 and 0.26 eV, while spin-flip LC-TDDFT tends to slightly underestimate them by 0.49 and 0.24 eV, for the monomer and dimer, respectively. The table also shows that the dimer provides large oscillator strength for the S$_2$ excitation, indicating the SF process from the S$_2$ excitation similar to that of the derivative (a), though the monomer gives the large oscillator strength only for the S$_1$ excitation. Assuming the experimentally-observed CT band as the $^1$(TT)$_1$ excitation as above, the $^5$(TT)$_1$ excitation energy is slightly underestimated but close to the experimental value. The MOs corresponding to the main transitions of the S$_1$ and S$_2$ excitations of the $\pi$-stacking TMBODIPY derivative (b) are illustrated in Fig. 4. The figure clearly shows that the S$_1$ excitation of the *pi*-stacking dimer consists of CT transitions, HOMO to LUMO+1 and HOMO−3 to LUMO, while the S$_2$ excitation of the monomer also includes a CT transition, HOMO−6 to LUMO, as shown in Fig. S3 of the supporting information. Note that these CT transitions are also obtained for cyclohexane. For this derivative, the calculated T$_1$ excitation energy (1.60 eV) is not close to the S$_1$ one (2.26 eV) for the dimer, while the T$_1$ excitation energy (1.42 eV) is much lower than the S$_2$ excitation energy (2.03 eV) for the monomer. These results also indicate that the ISC to the T$_1$ state may slowly proceed in parallel with the SF.

The triplet-state generation mechanism of the derivative (b) is, therefore, proposed similar to that of the derivative (a) as follows: After the S$_2$ excitation and the subsequent structural relaxation, the $\pi$-stacking dimer undergoes the spin-allowed transition to the $^1$(TT)$_1$ state in the vicinity of the intersection with the S$_2$ excitation. Then, the $^1$(TT)$_1$ state generates the long-lived mixing state with the $^5$(TT)$_1$ excitation by the triplet-exciton diffusion and subsequent re-encounter. The dimer in the $^5$(TT)$_1$ state is finally split into two triplet states. In conjunction with this SF, the ISC to the T$_1$ state may also slowly proceed even in the monomer.

Finally, we should notice that the considerable difference between the electronic spectra of the monomer and dimer indicates that the feasibility of SF is not determined only by the S$_1$ and T$_1$ excitation energies of the monomer in Eq. (2) because SF essentially proceeds in dimers that can be easily split into the monomers as conventional SF chromophores naturally forming $\pi$-stacking dimers.

**Table 2:** Calculated vertical excitation energies (eV) of TMBODIPY derivative (b) for the monomer and $\pi$-stacking dimer by SF-LC-TDBLYP, LC-TDBLYP (monomer) and TD$\omega$B97XD (dimer) with cc-pVTZ basis sets and CPCM solvent effect of acetonitrile.

Excited state	Exp.^a^		SF-LC-TDBLYP			LC-TDBLYP/TD$\omega$B97XD		
	eV	nm	Main transitions	eV	nm	eV	nm	*f*
**Monomer in acetonitrile**								
T$_1$				1.26	987	1.42	875	
S$_1$	2.50	496	$$\left\{ \begin{array}{l} \text{ H }\rightarrow \text{ L }~(0.97) \\ \text{ H }-3\rightarrow \text{ L }~(-0.12) \end{array} \right. $$	2.01	618	2.89	428	0.6557
S$_2$			$$\left\{ \begin{array}{l} \text{ H }-3\rightarrow \text{ L }~(-0.96) \\ \text{ H }-6\rightarrow \text{ L }~(0.13) \end{array} \right. $$	2.03	612	3.51	353	0.0048
$\user2{\pi }$**-Stacking dimer in acetonitrile**								
T$_1$				1.60	775	1.44	859	
$^5$(TT)$_1$	$\sim$3.1	$\sim$400				2.88	430	
S$_1$	2.50	496	$$\left\{ \begin{array}{l} \text{ H }\rightarrow \text{ L+1 }~(-0.68) \\ \text{ H }-3\rightarrow \text{ L }~(0.66) \end{array} \right. $$	2.26	548	2.76	449	0.1583
S$_2$			$$\left\{ \begin{array}{l} \text{ H }-7\rightarrow \text{ L }~(-0.50) \\ \text{ H }-6\rightarrow \text{ L }~(0.45) \end{array} \right. $$	3.30	376	2.96	419	0.8576
S$_3$			$$\begin{array}{l} \text{ H,H }\rightarrow \text{ L,L }~(-0.97) \end{array}$$	3.46	358	3.46	358	0.0154
S$_4$			$$\left\{ \begin{array}{l} \text{ H }-10\rightarrow \text{ L }~(0.74) \\ \text{ H }-8\rightarrow \text{ L }~(0.42) \end{array} \right. $$	3.62	342	3.47	357	0.1396

^a^Ref.^12^.

Main transitions of SF-LC-TDBLYP excitations are also shown with the coefficients of the response functions in parentheses, in which the notations H and L indicate HOMO and LUMO. The oscillator strengths (*f*) of LC-TDBLYP (monomer) and TD$\omega$B97XD (dimer) are also listed.

## Conclusions

In summary, we have theoretically investigated the role of SF in the triplet-state generations of TMBODIPY derivatives with electron acceptor and donor groups, (a) and (b). We, first, calculated the adsorption energies to the TMBODIPY derivatives for three types of solvent molecules, cyclohexane, chloroform and acetonitrile molecules, which are subtracted by the clustering energies of these solvent molecules. Comparing them with the $\pi$-stacking energies of these derivative molecules, we surprisingly found that the hierarchy of the adsorption energies and the $\pi$-stacking energies strongly correlates with the solvent dependence of the triplet-state generations in the experiment^15^, for five and more solvent molecules. In particular, the hierarchy for chloroform solvent is different depending on the derivatives in consistent with the experimental finding on the triplet-state generations. We, therefore, explored the feasibility of SF for the $\pi$-stacked TMBODIPY derivatives (a) and (b) in acetonitrile solvent by calculating their excitation spectra using spin-flip LC-TDDFT that one of the authors (Tsuneda) has developed. Consequently, we found that the S$_2$ excitations provide large oscillator strengths for both derivatives, and their excitation energies are close to the $^5$(TT)$_1$ excitation energies, which are approximately the same as the $^1$(TT)$_1$ excitation energies. These results strongly support that SF plays a main role in the triplet-state generations of these TMBODIPY derivatives. Note, however, that both these derivatives also undergo very slow ISCs in parallel with the SF, because they have low-lying excitations of CT characters which are advantageous to ISCs according to the El-Sayed rule. We, therefore, conclude that SF initiates the triplet-state generations of heavy atom-free organic photosensitizers such as BODIPY derivatives under the following two conditions:

1. Near-degenerate low-lying S and (TT) excitations with a considerable S-T energy gap, and
2. The moderate $\pi$-stacking energy of chromophores, which is higher than but not far from the solvation energy, for the dissociation generating triplet-state chromophores.

## Supplementary Information

Supplementary Information.
