# Supplementary material for: Singlet fission initiating triplet generations of BODIPY derivatives through π-stacking: a theoretical study
Source: Sci Rep. 2022 Nov 16;12:19714. doi: 10.1038/s41598-022-23370-y (PMC9668823; doi:10.1038/s41598-022-23370-y)

Supporting Information

Singlet fission initiating triplet generations of BODIPY derivatives through π-stacking: A theoretical study

Takao Tsuneda[*,a,b] and Tetsuya Taketsugu[a,c]

[a] Department of Chemistry, Faculty of Science, Hokkaido University, Sapporo 060-0810, Japan

[b] Graduate School of Science Technology and Innovation, Kobe University, Nada-ku, Kobe, Hyogo 657-8501, Japan

[c] Institute for Chemical Reaction Design and Discovery (WPI-ICReDD), Hokkaido University, Sapporo 001-0021, Japan

* Corresponding author: tsuneda@phoenix.kobe-u.ac.jp

Fig. S1. The optimized adsorption structures of solvent molecules, cyclohexane, chloroform and acetonitrile molecules, to the TMBODIPY derivatives (a) and (b), which are calculated using ωB97XD/cc-pVTZ method.

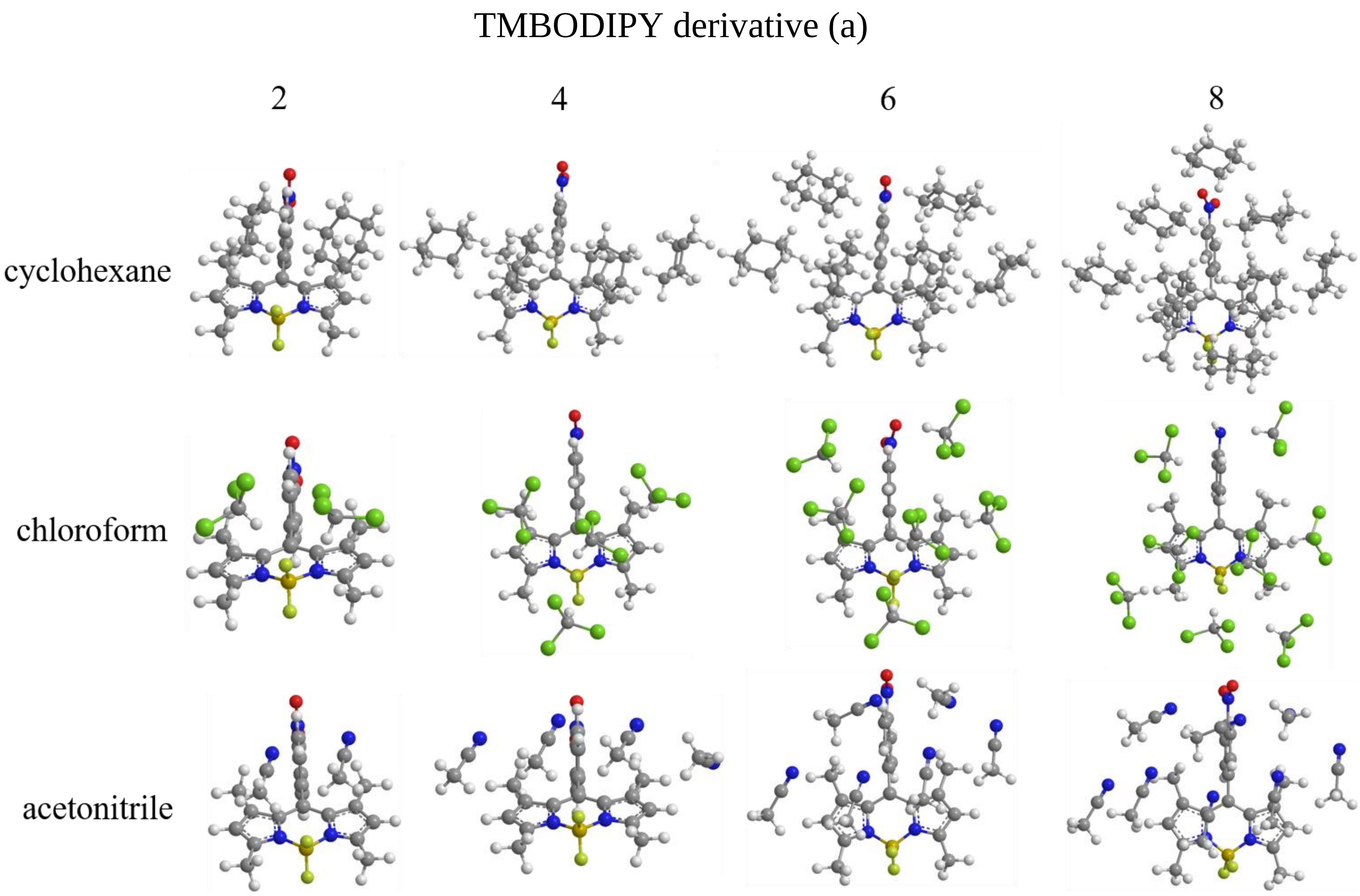

TMBODIPY derivative (b)

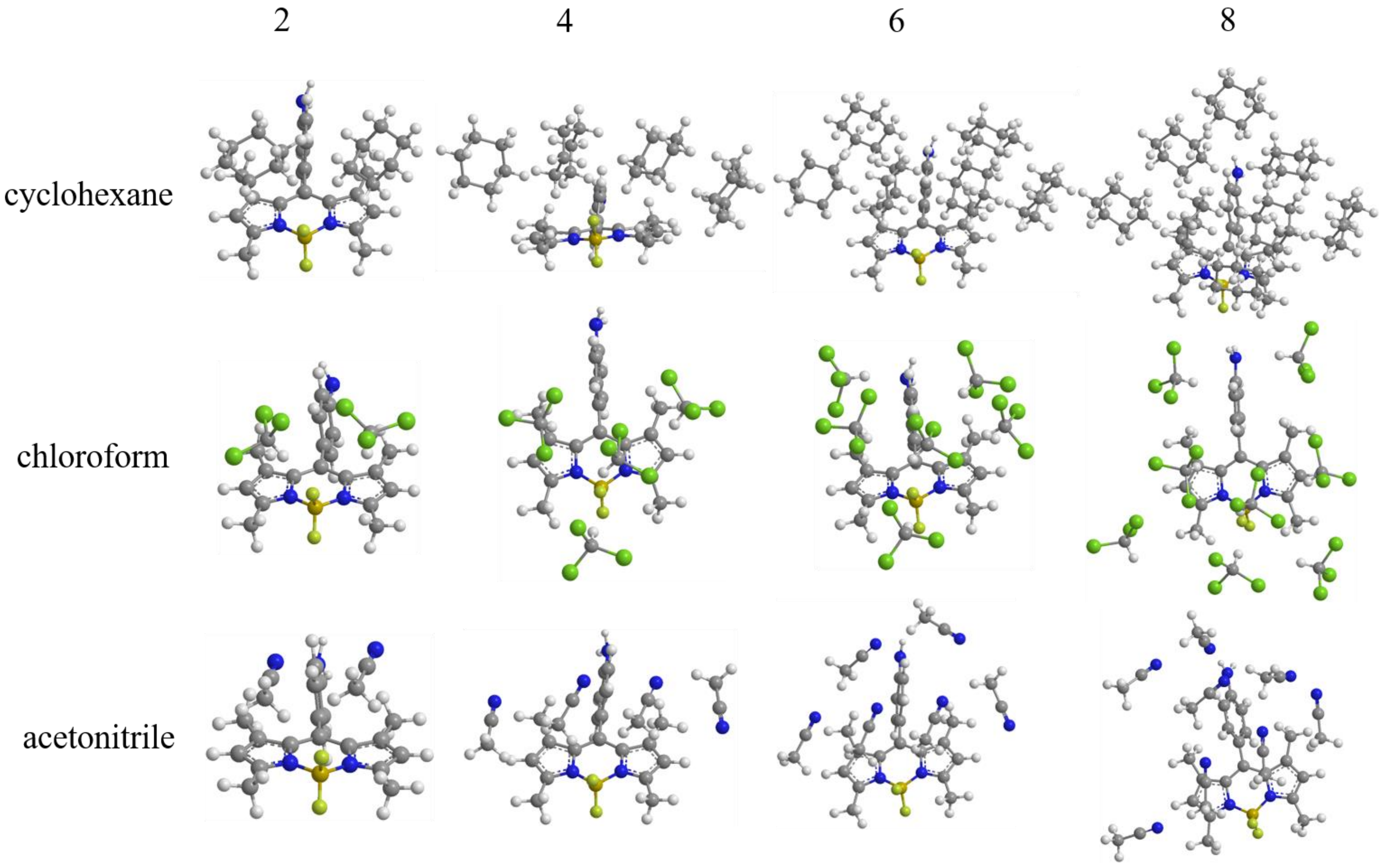

Fig. S2. The adsorption energies to TMBODIPY derivatives (a) and (b) and clustering energies of solvent molecules, cyclohexane, chloroform and acetonitrile molecules, which are calculated using ωB97XD/cc-pVTZ method. The optimized structures of the clusters of eight solvent molecules are also shown.

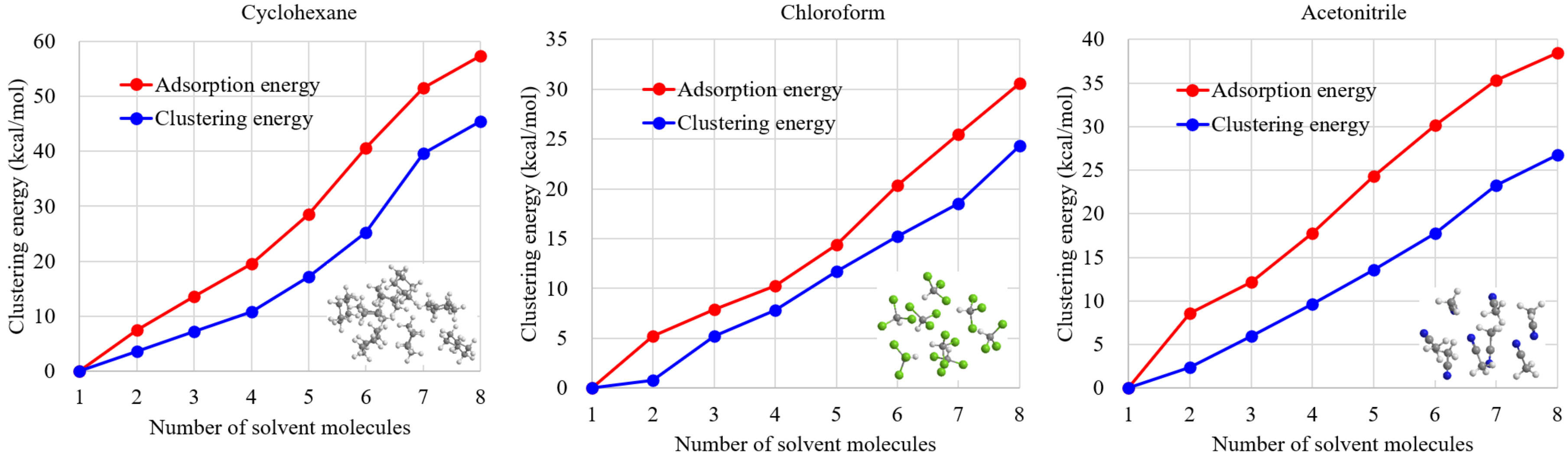

Fig. S3. The MO images corresponding to the main transitions of the S_1 and S_2 excitations of TMBODIPY derivatives (a) and (b) monomers and π-stacking dimers in SF-LC-TDBLYP/cc-pVTZ calculations.

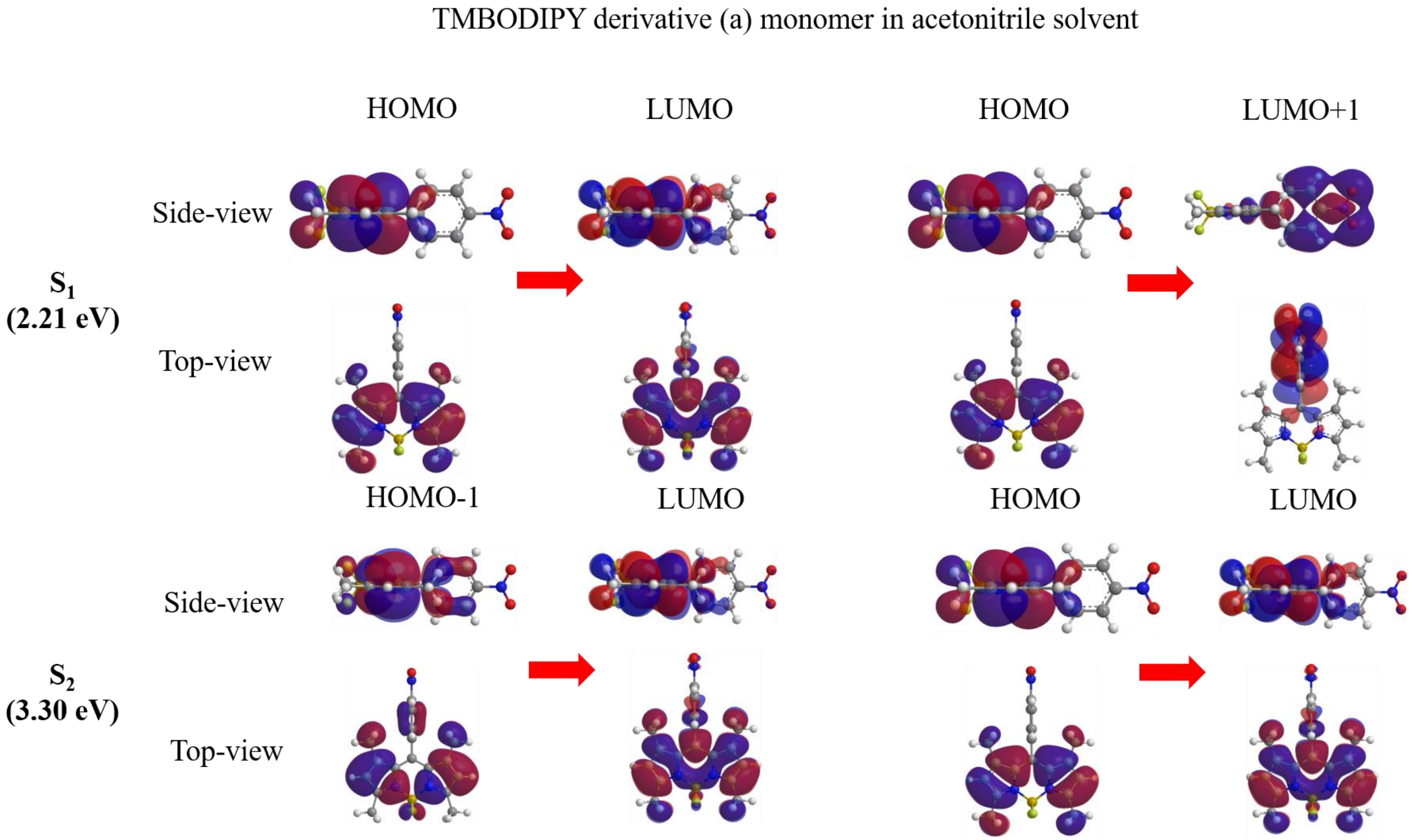

TMBODIPY derivative (b) monomer in acetonitrile solvent

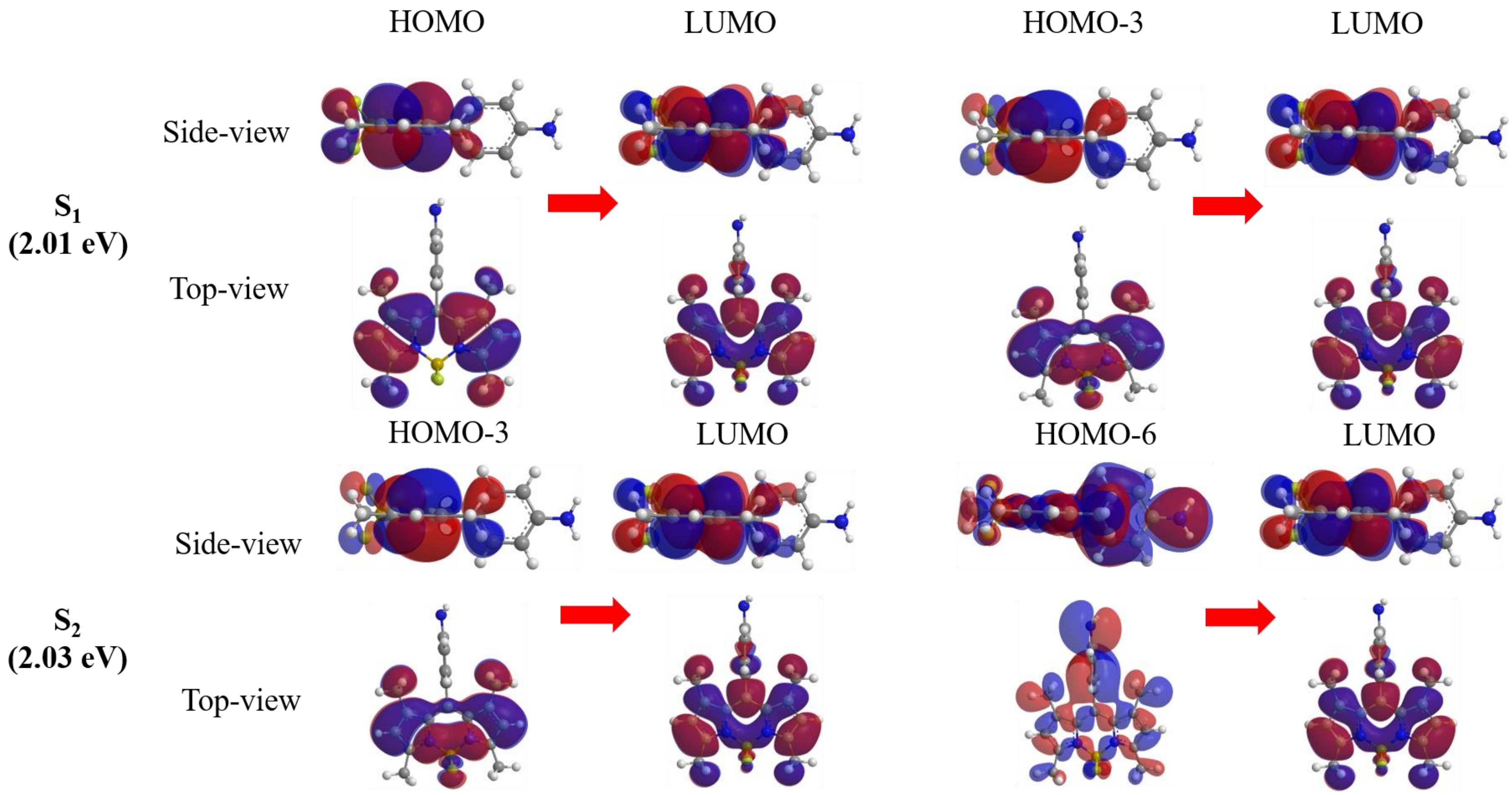

Fig. S4. The MO images corresponding to the main transitions of the S_3 and S_4 excitations of TMBODIPY derivatives (a) and (b) π-stacking dimers in SF-LC-TDBLYP/cc-pVTZ calculations.

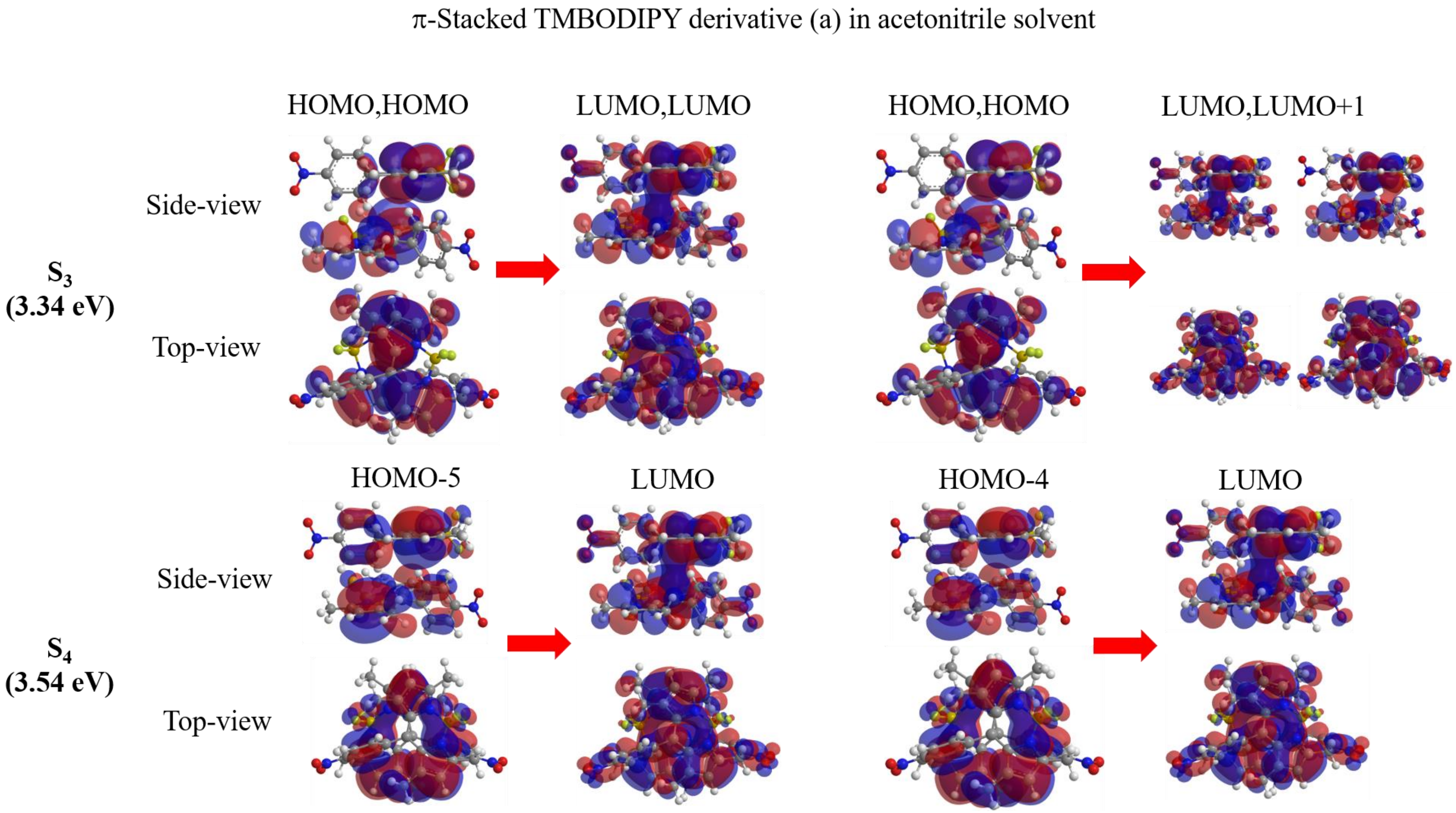

π-Stacked TMBODIPY derivative (b) in acetonitrile solvent

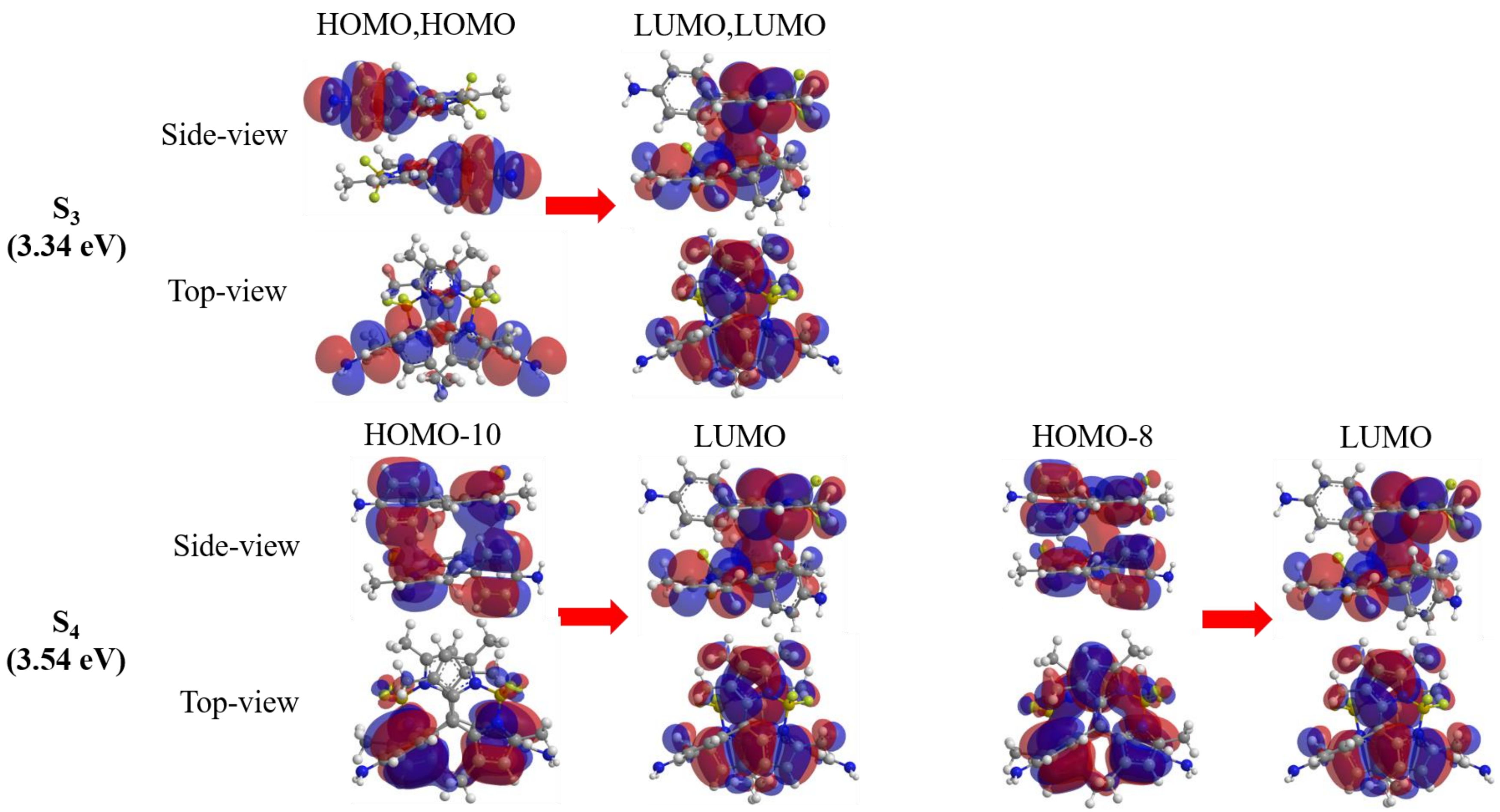

Supplement: Supplementary file 1 — Supplementary Information. [file 41598_2022_23370_MOESM1_ESM.pdf]
